# The ubiquitin ligase NEDD4-2/NEDD4L regulates both sodium homeostasis and fibrotic signaling to prevent end-stage renal disease

**DOI:** 10.1038/s41419-021-03688-7

**Published:** 2021-04-14

**Authors:** Jantina A. Manning, Sonia S. Shah, Andrej Nikolic, Tanya L. Henshall, Yeesim Khew-Goodall, Sharad Kumar

**Affiliations:** Centre for Cancer Biology, University of South Australia and SA Pathology, Adelaide, 5001 Australia

**Keywords:** Stress signalling, End-stage renal disease

## Abstract

Kidney disease progression can be affected by Na^+^ abundance. A key regulator of Na^+^ homeostasis is the ubiquitin ligase NEDD4-2 and its deficiency leads to increased Na^+^ transport activity and salt-sensitive progressive kidney damage. However, the mechanisms responsible for high Na^+^ induced damage remain poorly understood. Here we show that a high Na^+^ diet compromised kidney function in *Nedd4-2*-deficient mice, indicative of progression toward end-stage renal disease. Injury was characterized by enhanced tubule dilation and extracellular matrix accumulation, together with sustained activation of both Wnt/β-catenin and TGF-β signaling. *Nedd4-2* knockout in cortical collecting duct cells also activated these pathways and led to epithelial–mesenchymal transition. Furthermore, low dietary Na^+^ rescued kidney disease in *Nedd4-2*-deficient mice and silenced Wnt/β-catenin and TGF-β signaling. Our study reveals the important role of NEDD4-2-dependent ubiquitination in Na^+^ homeostasis and protecting against aberrant Wnt/β-catenin/TGF-β signaling in progressive kidney disease.

## Introduction

Chronic kidney disease (CKD), defined as a decline in kidney function over time, affects nearly 10% of the global population^1^. Mounting evidence suggests that excessive Na^+^ consumption can hasten the progression of CKD and contribute to end-stage renal disease (ESRD) via several mechanisms, including increased arterial pressure caused by hypertension^2^. High levels of Na^+^ can also influence CKD by compromising endothelial cell health and affecting tissue remodeling and fibrosis^3^.

The reabsorption of Na^+^ from dietary salt is facilitated by multiple channels and transporters in the kidney. The final stage of this process occurs in the distal tubule and the cortical collecting duct (CCD), controlled by the epithelial sodium channel (ENaC)^4^. The amount of functional ENaC on the plasma membrane is regulated by multiple factors including ubiquitination by the ubiquitin ligase NEDD4-2^5^. Genetic variation of *Nedd4L* (human *Nedd4-2*) is associated with developmental disorders, hypertension, and end-stage renal disease^6^. Furthermore, reduced expression of *Nedd4L* has been recently identified in diabetic nephropathy^7^, highlighting its relevance to kidney disease. Previously, kidney-specific deletion of *Nedd4-2* in mice has been shown to result in CKD-like pathology due to elevated ENaC levels driving increased uptake of Na^+^^8^. Feeding these mice a high Na^+^ diet during early postnatal stages results in aberrant ENaC regulation and increased kidney damage^9^. However, the mechanisms that are responsible for this Na^+^-sensitive renal damage remain unknown.

A number of signaling pathways have been implicated in the progression of CKD^10^. Wnt/β-catenin and TGF-β signaling^11^ are key events activated in response to high Na^+^^12,13^. TGF-β signaling, through activation of SMAD proteins, is a strong stimulator of extracellular matrix (ECM) production and fibrosis^12^. Similarly, sustained activation of Wnt/β-catenin signaling can drive CKD progression from acute kidney injury (AKI) by activating interstitial fibroblasts and ECM production^14^. Of note, the extent of Wnt signaling is important in influencing kidney disease, as it has also been shown to play a protective role in healing and repair^15^. Both of these signaling pathways are potent inducers of epithelial–mesenchymal transition (EMT)^16^, however significant debate as to the contribution of EMT-induced fibrosis to kidney disease has arisen in recent years^17^.

Here, we demonstrate that a short-term high Na^+^ diet fed to adult kidney-specific *Nedd4-2*-deficient mice promotes progression toward ESRD in the absence of hypertension. Sustained activation of Wnt/β-catenin and TGF-β signaling and resultant fibrotic deposition are key drivers of renal disease in mice. Similarly, these pathways are activated in *Nedd4-2*-deficient CCD cells in vitro, leading to EMT. Importantly, we show that a low Na^+^ diet ameliorates kidney disease in *Nedd4-2*-deficient mice by inhibiting Wnt/β-catenin and TGF-β signaling. We conclude that in addition to proper regulation of ENaC, NEDD4-2 mediated ubiquitination-dependent control of Wnt/β-catenin and TGF-β signaling is critical in preventing Na^+^-induced CKD progression.

## Results

### High dietary Na^+^ drives progression towards ESRD in *Nedd4-2*^*Ksp1.3*^ mice

**Fig. 1:**
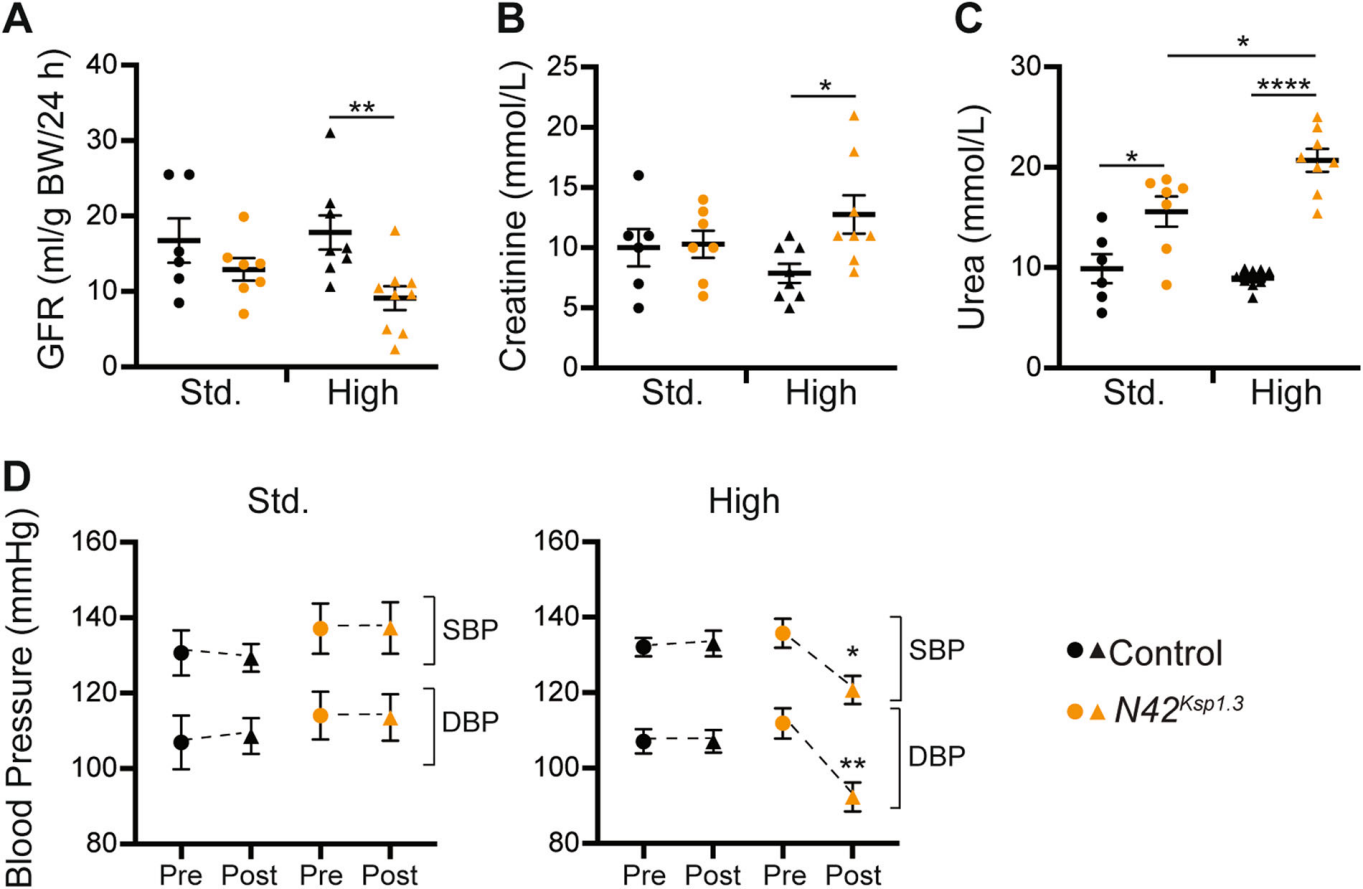
High dietary Na^+^ exacerbates renal disease in *Nedd4-2*^*Ksp1.3*^ mice, independently of hypertension. **A** GFR calculated from 24 h urine and blood collection after 17 days of standard (Std.) or high Na^+^ diet. **B**, **C** Serum creatinine and urea levels after varied diets. **D** BP measured by noninvasive tail-cuff averaged over 3 days for each time point. Pre: before the commencement of diet, Post: at the conclusion of varied Na^+^ diet. SBP systolic blood pressure, DBP diastolic blood pressure. **A**–**C** Significance determined using an unpaired two-tailed Student’s *t* test (means ± SEM). **D** Significance determined using one-way ANOVA of repeated measurements (means ± SEM, *n* = 9–11). ^*^P < 0.05, ^**^P < 0.01, ^****^P < 0.0001.

Mice deficient for *Nedd4-2* specifically in the renal tubules (*Nedd4-2*^*Ksp1.3*^) develop mild kidney disease due to upregulation of ENaC^8,9^. To evaluate the consequences of increased Na^+^ on kidney function, *Nedd4-2*^*Ksp1.3*^ adult mice were fed a standard Na^+^ (0.2%) or high Na^+^ (3.1%) diet for 17 days. Beyond this, significant weight loss required termination of the experiment. On the standard Na^+^ diet, there was no difference between the glomerular filtration rate (GFR) of control and *Nedd4-2*^*Ksp1.3*^ mice (Fig. 1A). However, after a high Na^+^ diet, *Nedd4-2*^*Ksp1.3*^ mice had a significant reduction in GFR. In addition, serum creatinine levels were significantly increased in *Nedd4-2*^*Ksp1.3*^ mice after high Na^+^ (Fig. 1B). Blood urea was increased on the standard Na^+^ diet, and further increased after high Na^+^ diet feeding (Fig. 1C). Hence, the underlying kidney injury present in *Nedd4-2*^*Ksp1.3*^ mice is exacerbated by high Na^+^ feeding, resulting in progression toward ESRD.

At 2 months of age, there was no significant difference in starting (Pre) blood pressure (BP) between control and *Nedd4-2*^*Ksp1.3*^ mice (Fig. 1D). On a standard Na^+^ diet, there was no change in the systolic BP or diastolic BP of mice of either genotype. Post high Na^+^ feeding, there was again no change in BP of control mice, however, *Nedd4-2*^*Ksp1.3*^ mice showed a significant decrease in BP (Fig. 1D). These results suggest that the reduction in kidney function caused by lack of *Nedd4-2* is not due to hypertension.

### Weight loss, polyuria and electrolyte imbalances are associated with renal disease

**Fig. 2:**
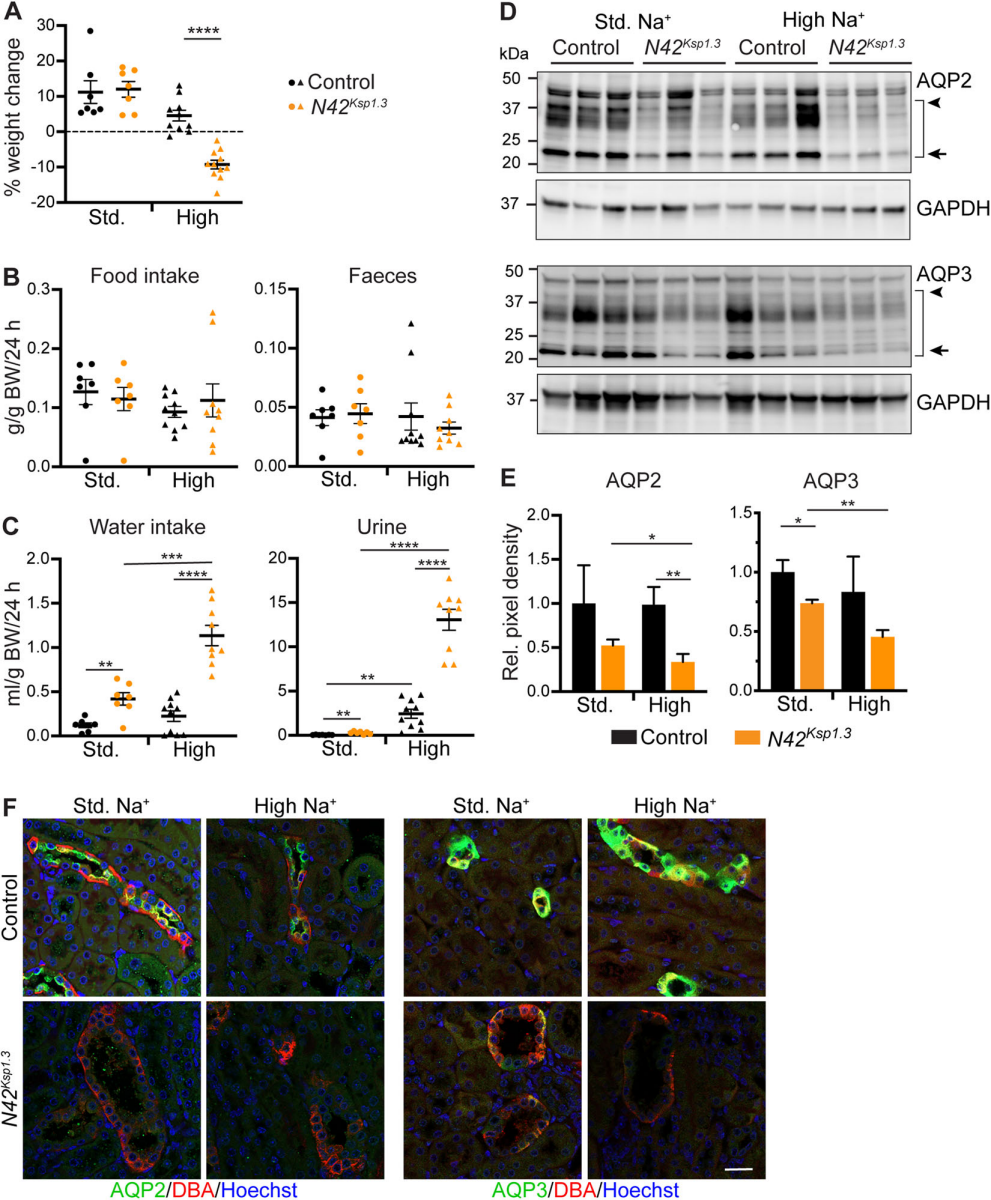
Weight loss, polyuria and AQP loss are associated with kidney disease in *Nedd4-2*^*Ksp1.3*^ mice. **A** Weight change at the conclusion of 17 days of the diet. **B** Food intake and feces produced or **C** water intake and urine output, over 24 h in metabolic cages. **D** Immunoblot analysis of AQP2 and AQP3 with GAPDH as a loading control. Arrow indicates unglycosylated form, arrowhead indicates highest glycosylated form. The area within the bracket represents quantitated region. **E** Quantitation of AQP immunoblots normalized to GAPDH loading control. **F** Immunostaining of AQP2 or AQP3 (green) with DBA marker for collecting ducts (red). DNA is stained by Hoechst (blue). Scale bar: 50 μm. Data presented as means ± SEM and significance determined using one-way ANOVA of repeated measurements (**A**), unpaired two-tailed Student’s *t* test (**B**, **C**), or presented as means ± SD with significance determined using unpaired two-tailed Student’s *t* test (**E**). *P < 0.05, **P < 0.01, ***P < 0.005, ****P < 0.0001.

*Nedd4-2*^*Ksp1.3*^ mice lost significant weight after high Na^+^ feeding (Fig. 2A). This loss primarily occurred after the first day of high Na^+^ (Supplementary Fig. 1). Food intake and feces output were similar, suggesting that the weight loss was not caused by a compromised appetite (Fig. 2B). *Nedd4-2*^*Ksp1.3*^ mice had a significant increase in water intake (polydipsia) and urine (polyuria), which was further increased after a high Na^+^ diet (Fig. 2C). In the collecting duct, Aquaporin-2 (AQP2) is the primary channel responsible for water reabsorption that allows concentration of urine, with loss of this channel causing polyuria^18^. Consistent with this, there was a trend toward a reduction in AQP2 (unglycosylated and glycosylated forms) in *Nedd4-2*^*Ksp1.3*^ kidneys on a standard Na^+^ diet, which was significantly reduced after high Na^+^ feeding (Fig. 2D, E). Similarly, AQP3 levels were reduced in *Nedd4-2*^*Ksp1.3*^ kidneys, and further reduced after high Na^+^ (Fig. 2D, E). Immunostaining of kidney sections revealed AQP2 localization in the apical membrane of primarily cortical collect duct cells, marked by *Dolichos Biflorus* Agglutinin (DBA) (Fig. 2F), as expected^19^. A dramatic reduction in AQP2 expression in *Nedd4-2*^*Ksp1.3*^ kidneys was observed in both diet conditions (Fig. 2F). AQP3 was greatly reduced in *Nedd4-2*^*Ksp1.3*^ kidneys on standard Na^+^ and further reduced by high Na^+^. These results suggest that water imbalance and osmotic stress are key features of salt-sensitive kidney disease in *Nedd4-2*-deficient mice.

In support of this, compared to control mice, urine of *Nedd4-2*^*Ksp1.3*^ mice had significantly decreased osmolarity and creatinine and increased urea levels after the high Na^+^ diet (Table 1). Despite the maintenance of urinary Na^+^, both K^+^ and Ca^2+^ levels were elevated. Blood serum levels of K^+^ and Cl^−^ were decreased, and Ca^2+^ increased in *Nedd4-2*^*Ksp1.3*^ mice after high Na^+^ compared to control mice (Table 1). Furthermore, additional indicators of kidney function including sodium bicarbonate (HCO_3_−), globulin, and total protein were increased in *Nedd4-2*^*Ksp1.3*^ mice. As previously reported in these mice^8^, significantly lower aldosterone levels were observed under standard Na^+^, further exacerbated by high dietary Na^+^.

**Table 1:** Urine and serum analysis of control and *Nedd4-2*^*Ksp1.3*^ mice on standard and high Na^+^ diets.

	Standard Na^+^		High Na^+^	
	Control	*Nedd4-2*^*Ksp1.3*^	Control	*Nedd4-2*^*Ksp1.3*^
*Urine*				
Osmolarity (mOsmol/kg)	1426.50 ± 380.60 (4)	698.00 ± 128.40 (7)	1701.33 ± 80.55 (6)	**550.78** ± 96.34**** (9)
Creatinine (Cr) (mM)	2.15 ± 0.42 (6)	0.99 ± 0.32 (7)	1.16 ± 0.23 (9)	**0.34** ± 0.19*** (10)
Urea/Cr	550.93 ± 28.32 (4)	461.53 ± 76.18 (5)	478.44 ± 52.46 (9)	**751.11** ± 82.45* (9)
Na^+^/Cr	53.01 ± 5.02 (4)	48.94 ± 7.80 (6)	635.0 ± 96.68 (9)	663.60 ± 90.66 (9)
K^+^/Cr	116.97 ± 8.52 (3)	105.16 ± 16.20 (6)	125.72 ± 8.40 (9)	**186.50** ± 18.21* (9)
Cl^−/^Cr	72.48 ± 5.54 (4)	59.50 ± 11.65 (6)	620.90 ± 89.16 (9)	622.20 ± 86.81 (9)
Ca^2+^/Cr	2.12 ± 1.21 (4)	4.40 ± 0.91 (6)	3.00 ± 0.61 (9)	**19.19** ± 2.42*** (9)
Protein/Cr	1326.94 ± 194.60 (5)	1422.14 ± 157.29 (7)	1441.56 ± 126.23 (9)	1041.67 ± 215.86 (9)
*Serum*				
Na^+^ (mM)	152.00 ± 0.55 (5)	151.29 ± 1.43 (7)	154.89 ± 2.12 (9)	153.63 ± 2.17 (8)
K^+^ (mM)	8.75 ± 0.60 (4)	7.56 ± 0.26 (7)	9.77 ± 0.69 (7)	**7.06** ± 0.35** (8)
Cl^-^ (mM)	105.00 ± 0.84 (5)	101.14 ± 1.49 (7)	115.44 ± 1.80 (9)	**103.75** ± 2.38** (8)
Ca^2+^ (mM)	2.77 ± 0.06 (5)	2.82 ± 0.04 (7)	2.46 ± 0.06 (9)	**2.80** ± 0.04*** (7)
HCO_3_^−^ (mM)	26.80 ± 0.97 (5)	26.14 ± .056 (7)	20.33 ± 1.11 (9)	**27.71** ± 2.20* (7)
Glucose (mM)	14.24 ± 1.16 (5)	14.24 ± 1.37 (7)	11.58 ± 0.33 (9)	11.81 ± 0.46 (8)
Globulin (g/L)	40.80 ± 0.80 (5)	42.00 ± 1.05 (7)	36.00 ± 1.28 (8)	**40.57** ± 0.65** (7)
Albumin (g/L)	16.00 ± 0.32 (5)	16.14 ± 0.67 (7)	14.67 ± 0.37 (9)	15.00 ± 0.38 (7)
Protein (g/L)	56.80 ± 1.02 (5)	58.14 ± 1.53 (7)	50.63 ± 1.60 (8)	**55.57** ± 0.90* (7)
Aldosterone (pmol/L)	234.50 ± 80.35 (4)	**10.17** ± 1.50* (5)	22.90 ± 3.66 (5)	**7.81** ± 1.21* (5)

Data presented as mean ± SEM for a number of mice (*n*) indicated in parentheses. Significance was determined using a Mann–Whitney test for non-normally distributed data, *Nedd4-2*^*Ksp1.3*^ vs. controls for each diet. ^*^P < 0.05, ^**^P < 0.01, ^***^P < 0.0005, ^****^P < 0.0001.

Bold values indicate statistical significance.

### Renal injury is characterized by ECM accumulation and fibrosis

**Fig. 3:**
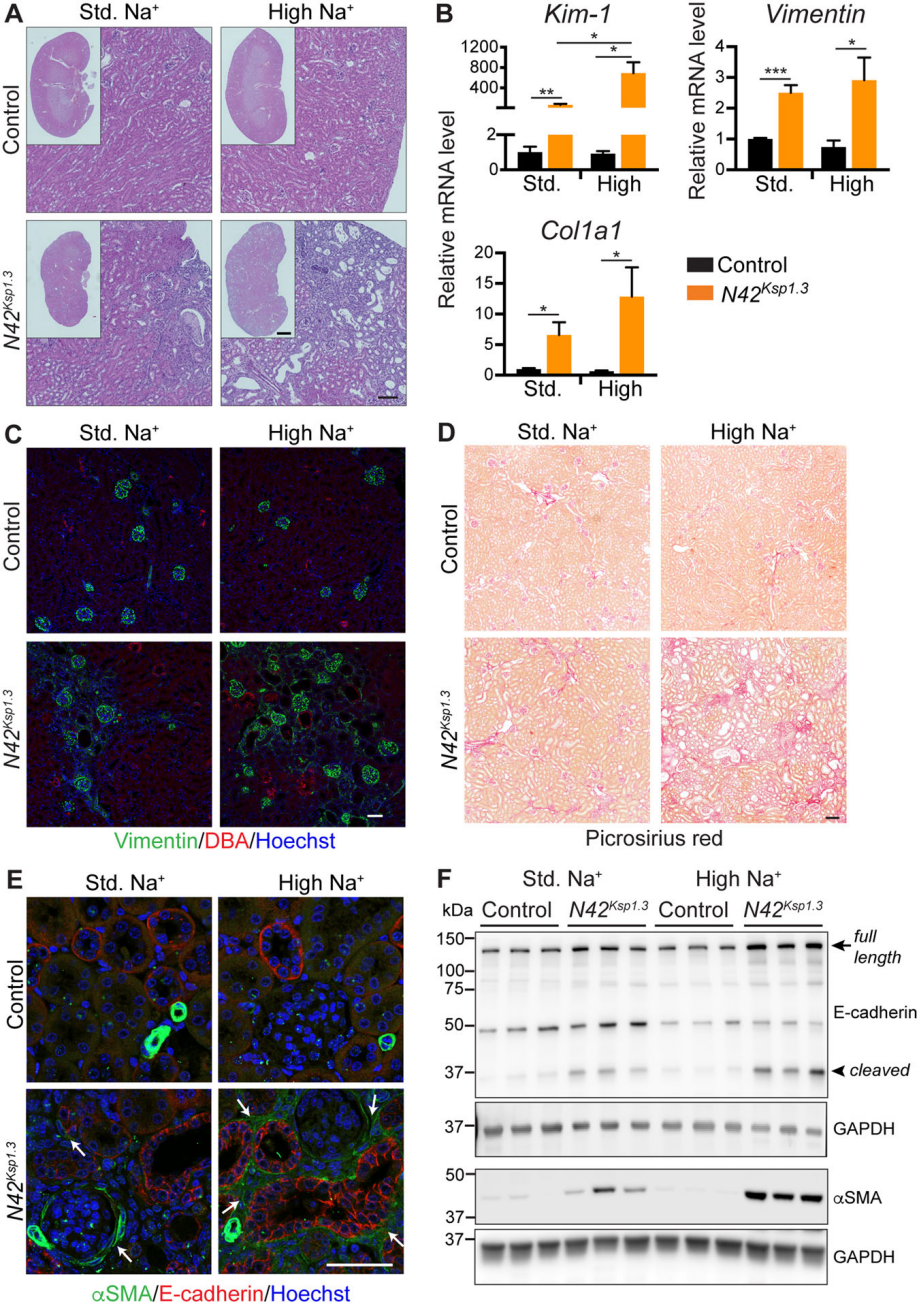
Kidney injury is characterized by ECM accumulation and fibrosis. **A** Haematoxylin and Eosin staining, scale bar: 100 μm, 1 mm for inset. **B** qRT-PCR analysis of *Kim-1*, *Vimentin*, and *Collagen 1* (*Col1a1*) relative to *TATA-box binding protein* (*TBP*). **C** Immunostaining of vimentin (green) with DBA marker for collecting ducts (red). DNA is stained by Hoechst (blue). Scale bar: 50 μm. **D** Picrosirius red staining of collagen, scale bar: 100 μm. **E** Immunostaining of αSMA (green) and E-cadherin (red). DNA is stained by Hoechst (blue). Arrows indicate regions of αSMA positive interstitium. Scale bar: 50 μm. **F** Immunoblot analysis of E-cadherin and αSMA with GAPDH as a loading control. Arrow indicates full-length E-cadherin, the arrowhead is 30 kDa cleaved form. Data in **B** presented as means ± SEM and significance determined using unpaired two-tailed Student’s *t* test, *n* = 3–4. ^*^P < 0.05, ^**^P < 0.01, ^***^P < 0.005.

*Nedd4-2*^*Ksp1.3*^ kidneys from mice fed a standard or high Na^+^ diet had an uneven appearance, as observed by gross morphology (Supplementary Fig. 2A). Haematoxylin and Eosin (H&E) staining of kidney sections revealed that the extent of injury in *Nedd4-2*^*Ksp1.3*^ kidneys consisting of dilated tubules, mesenchymal infiltration, and cellular debris was severely exacerbated after high Na^+^ (Fig. 3A, Supplementary Fig. 2B). The expression of *Kim-1*, a proximal tubule marker for kidney injury, was significantly increased in standard Na^+^ fed *Nedd4-2*^*Ksp1.3*^ mice and further increased by high Na^+^ (Fig. 3B). Additional markers of kidney injury, *Vimentin*, and *Collagen* (*Col1a1*) were increased in *Nedd4-2*^*Ksp1.3*^ *kidneys*, with similar levels after both diets (Fig. 3B, Supplementary Fig. 2C). An increase of ECM deposition and fibrosis after high Na^+^ was observed by vimentin immunostaining (Fig. 3C), and picrosirius red staining of connective tissue (Fig. 3D).

The myofibroblast marker, α-smooth muscle actin (αSMA), is normally present only surrounding the blood vessels in the kidney (Fig. 3E) but is upregulated during kidney injury^20^. Some αSMA positive cells were present surrounding the glomeruli and in the interstitium in *Nedd4-2*^*Ksp1.3*^ kidneys, which was apparent to a much greater extent following the high Na^+^ diet (Fig. 3E). This staining was distinct from E-cadherin positive epithelial tubular cells, suggesting a mesenchymal nature of these cells (Fig. 3E and lower magnification in Supplementary Fig. 2D). Loss of E-cadherin, a classical Ca^2+^-dependent cell adhesion molecule, is known to been associated with EMT and fibrosis. In contrast, here *Nedd4-2*^*Ksp1.3*^ mice had increased tubular basolateral E-cadherin staining which was more widespread following high Na^+^ with some staining on the apical membrane, suggesting a loss of polarity (Fig. 3E). Similarly, the total protein levels of αSMA and E-cadherin were elevated in *Nedd4-2*^*Ksp1.3*^ kidneys, and to a greater extent by the high Na^+^ diet (Fig. 3F, quantitated in Supplementary Fig. 2E). Interestingly, an increase in a 30 kDa E-cadherin fragment was observed in *Nedd4-2*^*Ksp1.3*^ kidneys (Fig. 3F), similar to that previously reported in the ligated kidney^21^.

### Damage to parenchyma involves sustained activation of Wnt/β-catenin and TGF-β signaling

**Fig. 4:**
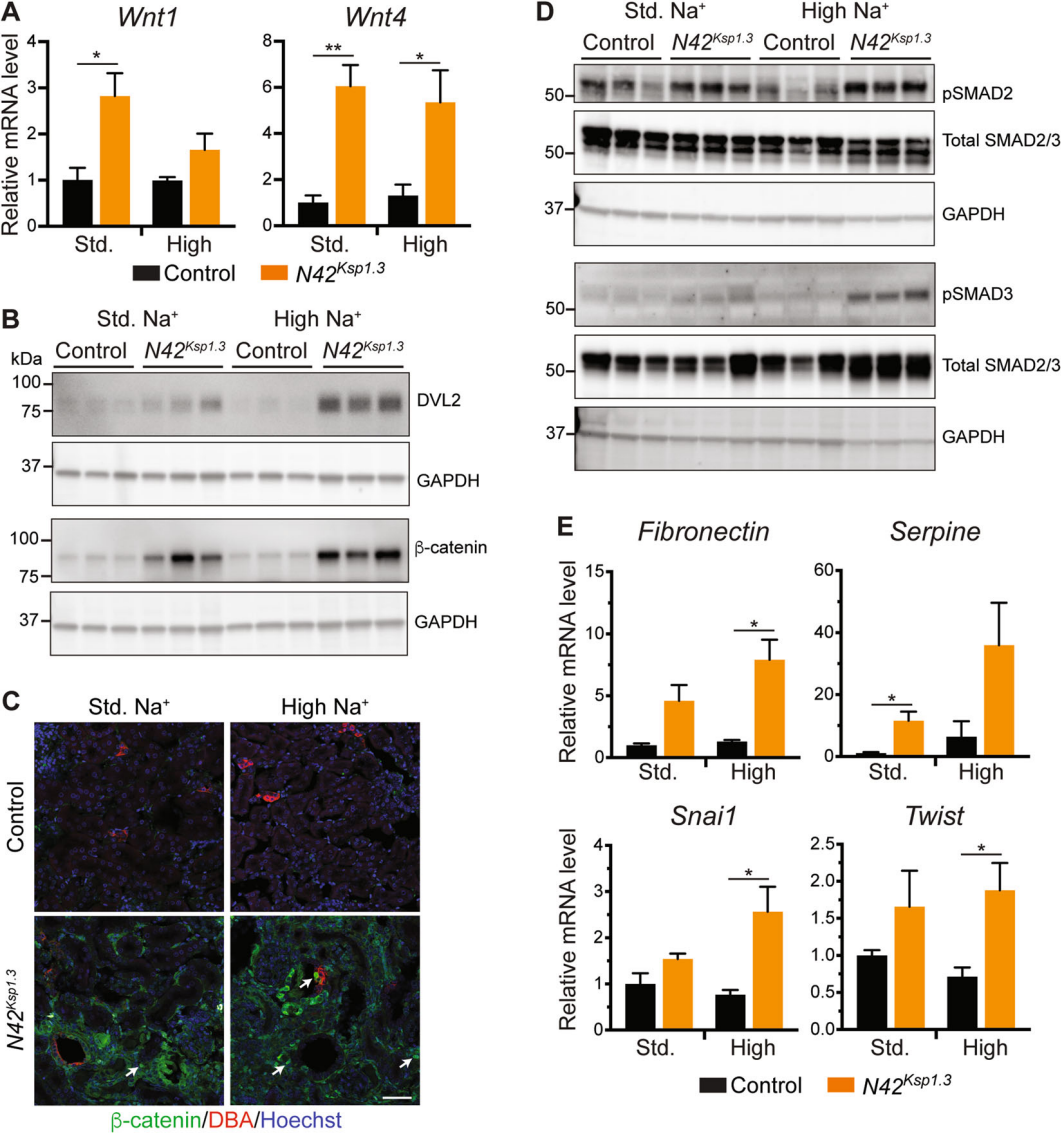
Damage to parenchyma involves sustained activation of Wnt/β-catenin and TGF-β signaling. **A** qRT-PCR analysis of *Wnt1* and *Wnt4* genes relative to the *TBP* housekeeping gene. **B** Immunoblot analysis of β-catenin and DVL2 with GAPDH as a loading control. **C** Immunostaining of β-catenin (green) with DBA marker for collecting ducts (red). DNA is stained by Hoechst (blue). Arrows indicate nuclear staining. Scale bar: 50 μm. **D** Immunoblot analysis of pSMAD2/3 and total SMAD2/3 with GAPDH as a loading control. **E** qRT-PCR analysis of genes relative to *TBP*. Data presented as means ± SEM and significance determined using unpaired two-tailed Student’s *t* test, *n* = 3–4. ^*^P < 0.05, ^**^P < 0.01.

As Wnt and TGF-β signaling are well known to play a role in CKD progression^22^, we examined if kidney injury in *Nedd4-2*-deficient mice is caused by defective Wnt and TGF-β signaling. The expression of *Wnt1*, which is activated early after kidney injury^14^ was upregulated in *Nedd4-2*^*Ksp1.3*^ kidneys on the standard Na^+^ diet, however, was comparable to controls following the high Na^+^ diet (Fig. 4A). A similar pattern was observed for *Wnt3* (Supplementary Fig. 3A). *Wnt4*, which has been demonstrated to have sustained upregulation after severe kidney injury, was upregulated in *Nedd4-2*^*Ksp1.3*^ kidneys on the standard Na^+^ diet and maintained after high Na^+^ (Fig. 4A). NEDD4-2 has been implicated in Wnt signaling by promoting Disheveled 2 (DVL2) ubiquitination and degradation leading to inhibition of β-catenin degradation^22,23^. Levels of DVL2 were increased in *Nedd4-2*^*Ksp1.3*^ kidneys on standard Na^+^ and further after high Na^+^ diet (Fig. 4B, quantitated in Supplementary Fig. 3B). Consistently, *Nedd4-2*^*Ksp1.3*^ kidneys had increased total β-catenin on standard and high Na^+^ diets (Fig. 4B). Immunostaining revealed an accumulation of cytoplasmic β-catenin in *Nedd4-2*^*Ksp1.3*^ kidneys after both diets, with evidence of translocation into the nucleus (Fig. 4C). This was not restricted to DBA-positive collecting ducts which lack NEDD4-2 expression, suggesting a nonautonomous role of *Nedd4-2*-deficiency in activating this signaling pathway.

In addition to Wnt, TGF-β signaling has well-established links to CKD, with TGF-βR1 and SMAD2/3 components of this pathway regulated by NEDD4-2-dependent ubiquitination^24,25^. Whilst no increase in TGF-β1 ligand, or its receptor, TGF-βR1, was observed in *Nedd4-2*^*Ksp1.3*^ kidneys (Supplementary Fig. 3A, C), levels of phosphorylated (p) SMAD2 and SMAD3, known NEDD4-2 substrates^24^, were increased on the standard Na^+^ diet, and pSMAD2 was further increased after high Na^+^ (Fig. 4D, quantitated in Supplementary Fig. 3B). Increased expression of *Fibronectin* and *Serpine*, two Wnt/TGF-β target genes involved in the fibrotic response^15^, were observed in *Nedd4-2*^*Ksp1.3*^ kidneys, reaching significance on either standard Na^+^ diet (*Serpine*), or after high Na^+^ (*Fibronectin*) (Fig. 4E). However, targets genes of the cell cycle and proliferation, *cMyc* and *Cyclin D1* were not changed (Supplementary Fig. 3D). Interestingly, genes promoting EMT in kidney disease, *Snai1*, and *Twist* were significantly increased in *Nedd4-2*^*Ksp1.3*^ kidneys only after high Na^+^, suggesting that EMT may play a role in the severity of this disease (Fig. 4E).

### Nedd4-2 KO in CCD cells confirms the direct role of NEDD4-2 in Wnt/β-catenin/TGF-β signaling

**Fig. 5:**
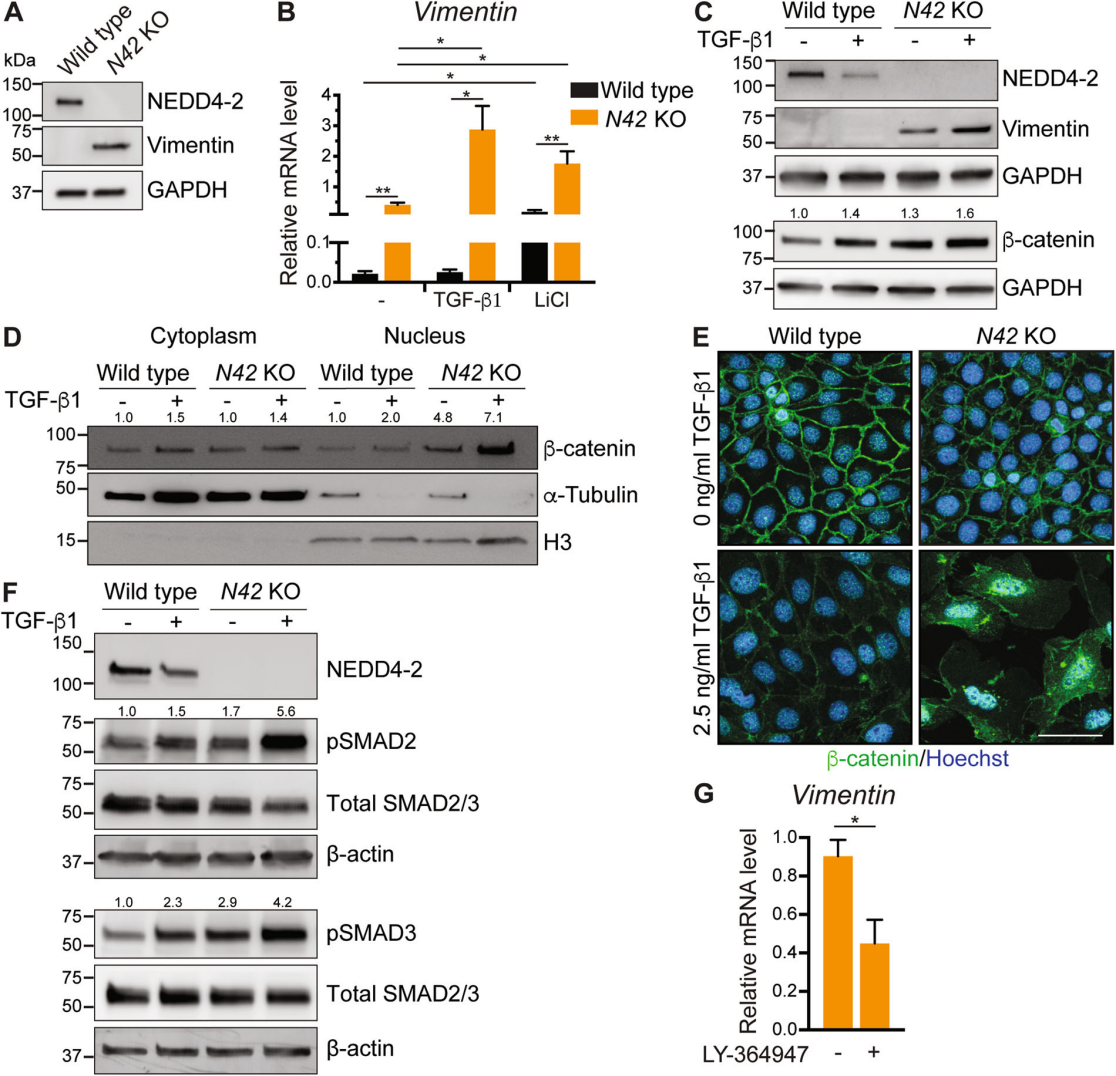
NEDD4-2 is implicated in Wnt/β-catenin/TGF-β signaling in CCD cells. CRISPR–Cas9 was used to generate CCD-N21 clones lacking NEDD4-2 or wild-type control clones. **A** Immunoblot analysis of NEDD4-2 and Vimentin with GAPDH as a loading control. **B** qRT-PCR analysis of *Vimentin* relative to *TBP* in untreated cells, 2.5 ng/ml TGF-β1 or 10 mM LiCl treated for 72 h, *n* = 4 for 4 independent clones each. **C** Immunoblot analysis of NEDD4-2, vimentin, and β-catenin with GAPDH as a loading control, untreated or after 72 h TGF-β1 stimulation. Numbers indicate quantitation of β-catenin bands relative to untreated wild type lane, normalized to GAPDH. **D** Immunoblot analysis of β-catenin after separation of cytoplasmic (marked by α-tubulin) and nuclear (marked by Histone 3, H3) fractions. Numbers indicate quantitation of β-catenin bands relative to untreated wild-type lane, normalized to α-tubulin for a cytoplasmic fraction or H3 for nuclear fraction. **E** Immunostaining of β-catenin (green) with and without TGF-β1 treatment. DNA is stained by Hoechst (blue). Scale bar: 50 μm. **F** Immunoblot analysis of pSMAD2/3 and total SMAD2/3 with GAPDH as a loading control. Numbers indicate quantitation of pSMAD bands relative to untreated wild-type lane, normalized to β-actin. **G** qRT-PCR analysis of *Vimentin* relative to *TBP* in untreated or 72 h 500 nM LY-364947 treated *Nedd4-2* KO cells, *n* = 4. Data presented as means ± SEM (**B**, **G**) and significance determined using unpaired two-tailed Student’s *t* test. ^*^P < 0.05, ^**^P < 0.01. Immunoblots are representative of three experiments with similar results.

To further investigate the roles of Wnt/β-catenin and TGF-β signaling in kidney disease resulting from *Nedd4-2* deficiency, we generated *Nedd4-2* knockout (KO) in a CCD cell line. Sequencing confirmed a frameshift mutation (Supplementary Fig. 4A), that resulted in a complete loss of NEDD4-2 protein expression (Fig. 5A, Supplementary Fig. 4B) and increased vimentin (Fig. 5A, B). *Nedd4-2* KO resulted in increased sensitivity to Wnt and TGF-β signaling as treatment with TGF-β ligand or the Wnt activator LiCl^26^ further increased *Vimentin* expression (Fig. 5B). Interestingly, TGF-β exposure resulted in reduced endogenous NEDD4-2 levels as well as increased vimentin protein (Fig. 5C). Similar to *Nedd4-2*^*Ksp1.3*^ mice, β-catenin levels were elevated in *Nedd4-2* KO cells (Fig. 5C). β-catenin was increased in the nuclear fraction of *Nedd4-2* KO cells and was localized to the nucleus as well as the cell membrane, particularly after TGF-β stimulation (Fig. 5D, E and Supplementary Fig. 4C). DVL2 levels were unchanged (Supplementary Fig. 4D) indicating that the increase in β-catenin is independent of DVL2-dependent Wnt signaling. In addition, pSMAD2 and 3 levels were increased in KO cells and this was exacerbated by TGF-β treatment (Fig. 5F). Inhibition of TGF-βR1 using LY-364947 significantly reduced *Vimentin* levels in *Nedd4*-2 KO cells (Fig. 5G), even after stimulation with TGF-β1 (Supplementary Fig. 4E), confirming the contribution of TGF-β signaling to the *Nedd4-2* KO phenotype.

**Fig. 6:**
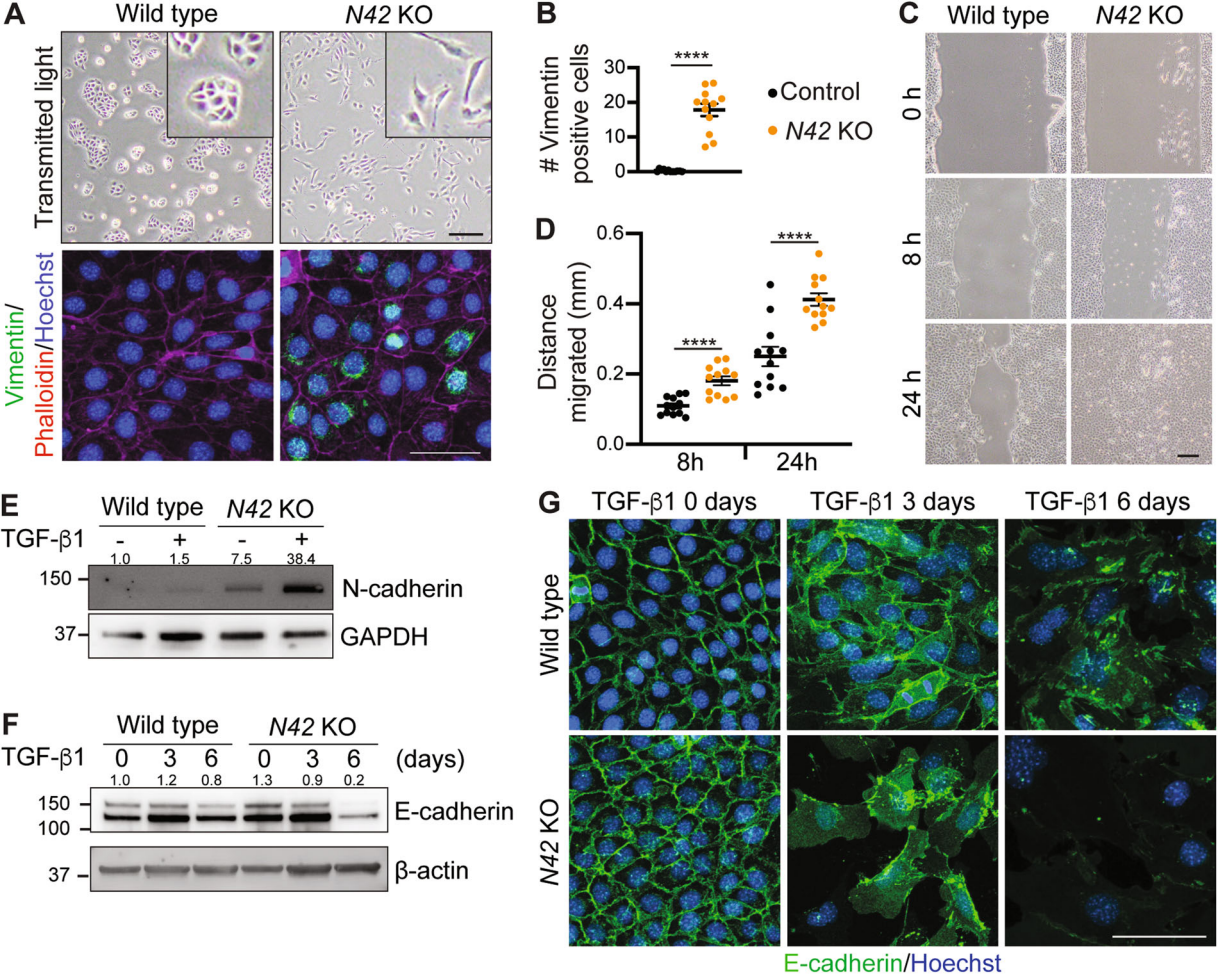
*Nedd4-2* KO CCD cells display EMT characteristics. **A** Light microscopy of wild type and *Nedd4-2* KO cells, scale bar: 200 μm (with higher magnification inset) or immunostaining of vimentin (green) and Phalloidin (red) with Hoechst staining of DNA (blue). Scale bar: 50 μm. **B** Number of vimentin-positive cells per field of view, *n* = 4 clones, each with three fields of view. **C** Light microscopy of cells showing migration distance after scratch, scale bar: 200 μm. **D** Migration distance quantitated in four clones, *n* = 3 replicates each. **E** Immunoblot analysis of N-cadherin with GAPDH as a loading control, untreated or after 72 h 2.5 ng/ml TGF-β1 stimulation. Numbers indicate quantitation of N-cadherin bands relative to untreated wild-type lane, normalized to GAPDH. **F** Immunoblot analysis of E-cadherin with β-actin as a loading control, untreated or after 3 or 6 days of TGF-β1 stimulation. Numbers indicate quantitation of E-cadherin bands relative to untreated wild type lane, normalized to β-actin. **G** Immunostaining of E-cadherin (green) after 0, 3, and 6 days of TGF-β1 treatment. DNA is stained by Hoechst (blue). Scale bar: 50 μm. Data presented as means ± SEM (**B**, **D**) and significance determined using unpaired two-tailed Student’s *t* test. ^****^P < 0.0001.

We next analyzed *Nedd4-2* KO cells for evidence of EMT as Wnt/β-catenin and TGF-β signaling are implicated in this process. Wild-type cells displayed a classic epithelial cobblestone morphology, whereas *Nedd4-2* KO cells were elongated with cellular protrusions, often positive for vimentin expression (Fig. 6A, B). Vimentin has been shown to promote cellular migration and a migratory phenotype is a characteristic of mesenchymal cells^27^. Consistent with this, *Nedd4-2* KO CCD cells demonstrated significantly increased migration compared to wild-type cells (Fig. 6C, D). In addition, *Nedd4-2* KO cells displayed an increase in N-cadherin, further exacerbated by TGF-β1 stimulation (Fig. 6E). Total E-cadherin levels remained stable after 3 days, although cytoplasmic accumulation was observed in response to the dissolution of adherens junctions (Fig. 6F, G). A further 3 days of TGF-β1 treatment showed a marked reduction in E-cadherin protein abundance and membrane localization. Together, these data demonstrate that loss of NEDD4-2 in CCD cells activates Wnt/β-catenin and TGF-β signaling, which is sufficient to drive partial EMT.

### Low Na^+^ diet inhibits Wnt/β-catenin/TGF-β signaling and kidney disease in *Nedd4-2*^*Ksp1.3*^ mice

**Fig. 7:**
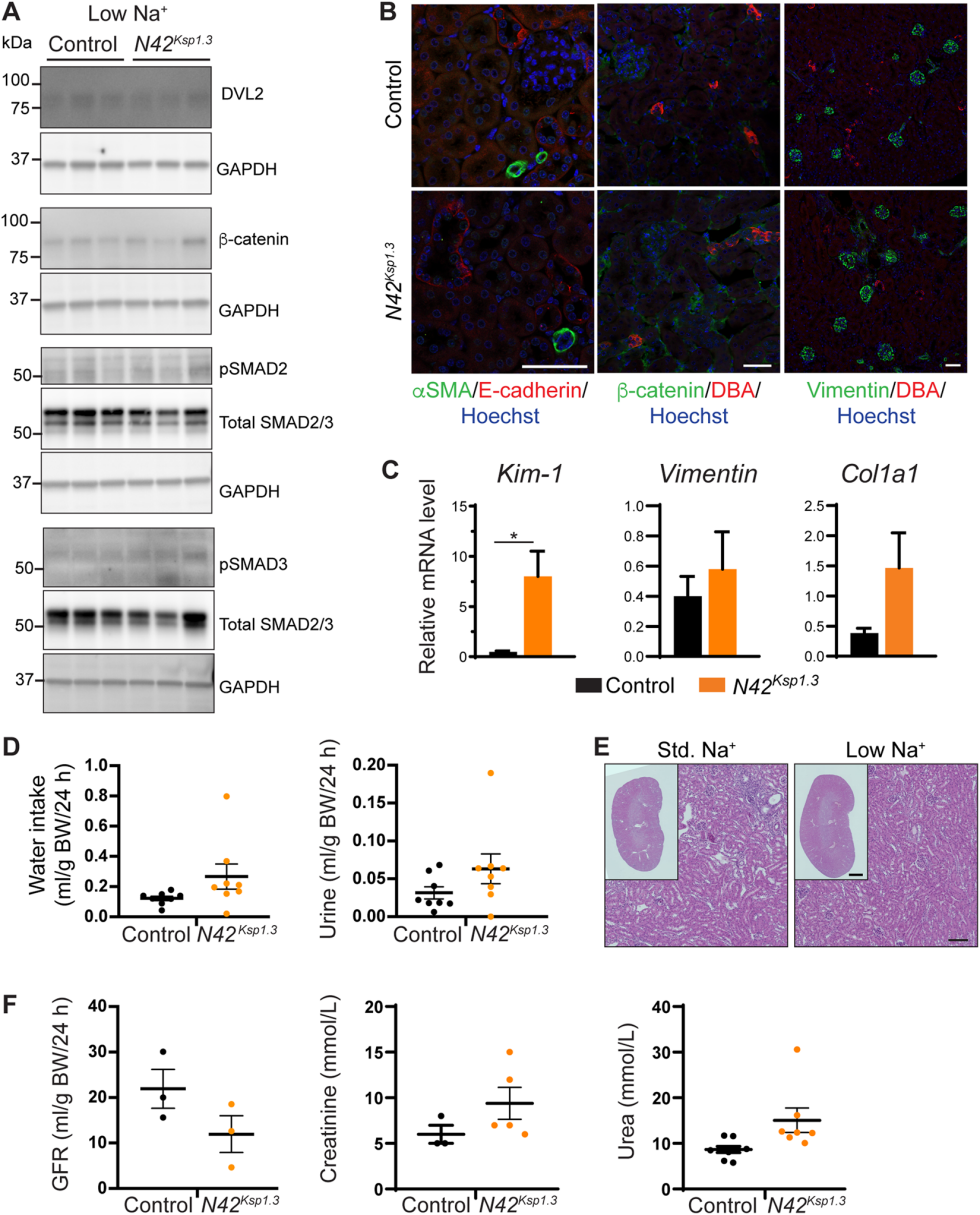
Low dietary Na^+^ ameliorates Wnt/β-catenin/TGF-β signaling and kidney disease in *Nedd4-2*^*Ksp1.3*^ mice. Mice were fed a low Na^+^ (0.05%) diet for 17 days. **A** Immunoblot analysis of DVL2, β-catenin, pSMAD2/3, and total SMAD2/3 with GAPDH as loading controls. **B** Immunostaining of αSMA (green) with E-cadherin (red), β-catenin (green) with DBA (red), and vimentin (green) with DBA (red). DNA is stained by Hoechst (blue). Scale bar: 50 μm. **C** qRT-PCR analysis of genes relative to *TBP*. **D** Water intake and urine output over 24 h in metabolic cages. **E** Haematoxylin and Eosin staining of kidneys, scale bar: 100 μm, 1 mm for inset. **F** GFR calculated from 24 h urine and blood collection, and serum creatinine and urine levels. Data presented as means ± SEM and significance determined using unpaired two-tailed Student’s *t* test, *n* = 3-4. ^*^P < 0.05.

To investigate whether low dietary Na^+^ could rescue Wnt/β-catenin/TGF-β signaling and kidney damage in adult mice, a low Na^+^ (0.05%) diet was fed to adult mice after the renal disease had been established. Levels of DVL2, β-catenin, pSMAD2, and pSMAD3 were similar in control and *Nedd4-2*^*Ksp1.3*^ kidneys (Fig. 7A). Similarly, immunostaining showed comparable levels of E-cadherin, αSMA, and β-catenin in *Nedd4-2*^*Ksp1.3*^ and control kidneys after the low Na^+^ diet (Fig. 7B). Correlating with reduced collagen and ECM deposition, vimentin was no longer increased (Fig. 7B). Whilst *Kim-1* expression was still increased in *Nedd4-2*^*Ksp1.3*^ kidneys, this was to a much lower extent when compared with mice fed a low Na^+^ diet (Figs. 7C and 3B). *Vimentin* and *Collagen* levels were no longer significantly different from controls. Importantly, polydipsia and polyuria were no longer present (Fig. 7D), and histological analysis revealed correction of renal pathology (Fig. 7E). GFR was not significantly different between control and *Nedd4-2*^*Ksp1.3*^ mice. Blood creatinine and urea levels were similar in both genotypes (Fig. 7F), with no other serum differences in electrolytes or kidney function (Table 2). Other than decreased creatinine and increased Ca^2+^, urine electrolyes were stablized. Collectively, these data demonstrate that kidney disease in *Nedd4-2*^*Ksp1.3*^ mice is rescued by low dietary Na^+^, due at least in part to the inhibition of Wnt/β-catenin and TGF-β signaling.

**Table 2:** Urine and serum analysis of control and *Nedd4-2*^*Ksp1.3*^ mice on low Na^+^ diet.

Low Na^+^		
	Control	*Nedd4-2*^*Ksp1.3*^
*Urine*		
Osmolarity (mOsmol/kg)	1325.67 ± 207.54 (3)	1125.25 ± 375.00 (4)
Creatinine (Cr) (mM)	4.09 ± 0.43 (9)	**1.86** ± 0.28 (7)**
Urea/Cr	532.75 ± 42.96 (8)	559.25 ± 94.06 (4)
Na^+^/Cr	52.75 ± 8.89 (8)	77.00 ± 11.83 (4)
K^+^/Cr	115.14 ± 9.88 (8)	138.88 ± 17.10 (4)
Cl^−^/Cr	64.63 ± 9.52 (8)	91.25 ± 11.92 (4)
Ca^2+^/Cr	0.83 ± 0.13 (8)	**4.27** ± 0.93* (4)
Protein/Cr	1199.63 ± 163.98 (8)	1216.50 ± 231.60 (4)
*Serum*		
Na^+^ (mM)	151.44 ± 0.96 (9)	152.29 ± 1.38 (7)
K^+^ (mM)	8.40 ± 0.24 (9)	8.93 ± 0.40 (7)
Cl^−^ (mM)	104.67 ± 0.60 (9)	103.57 ± 1.17 (7)
Ca^2+^ (mM)	2.66 ± 0.03 (9)	2.60 ± 0.06 (7)
HCO_3_^−^ (mM)	24.56 ± 0.82 (9)	22.42 ± 1.45 (7)
Glucose (mM)	12.88 ± 0.34 (9)	12.83 ± 0.42 (7)
Globulin (g/L)	39.78 ± 0.64 (9)	41.14 ± 0.63 (7)
Albumin (g/L)	16.00 ± 0.37 (9)	16.86 ± 0.51 (7)
Protein (g/L)	55.78 ± 0.86 (9)	58.00 ± 0.98 (7)
Aldosterone (pmol/L)	99.39 ± 25.82 (5)	49.34 ± 11.12 (4)

Data presented as mean ± SEM for a number of mice (*n*) indicated in parentheses. Significance was determined using a Mann–Whitney test for non-normally distributed data. ^*^P < 0.05, ^**^P < 0.01.

Bold values indicate statistical significance.

## Discussion

**Fig. 8:**
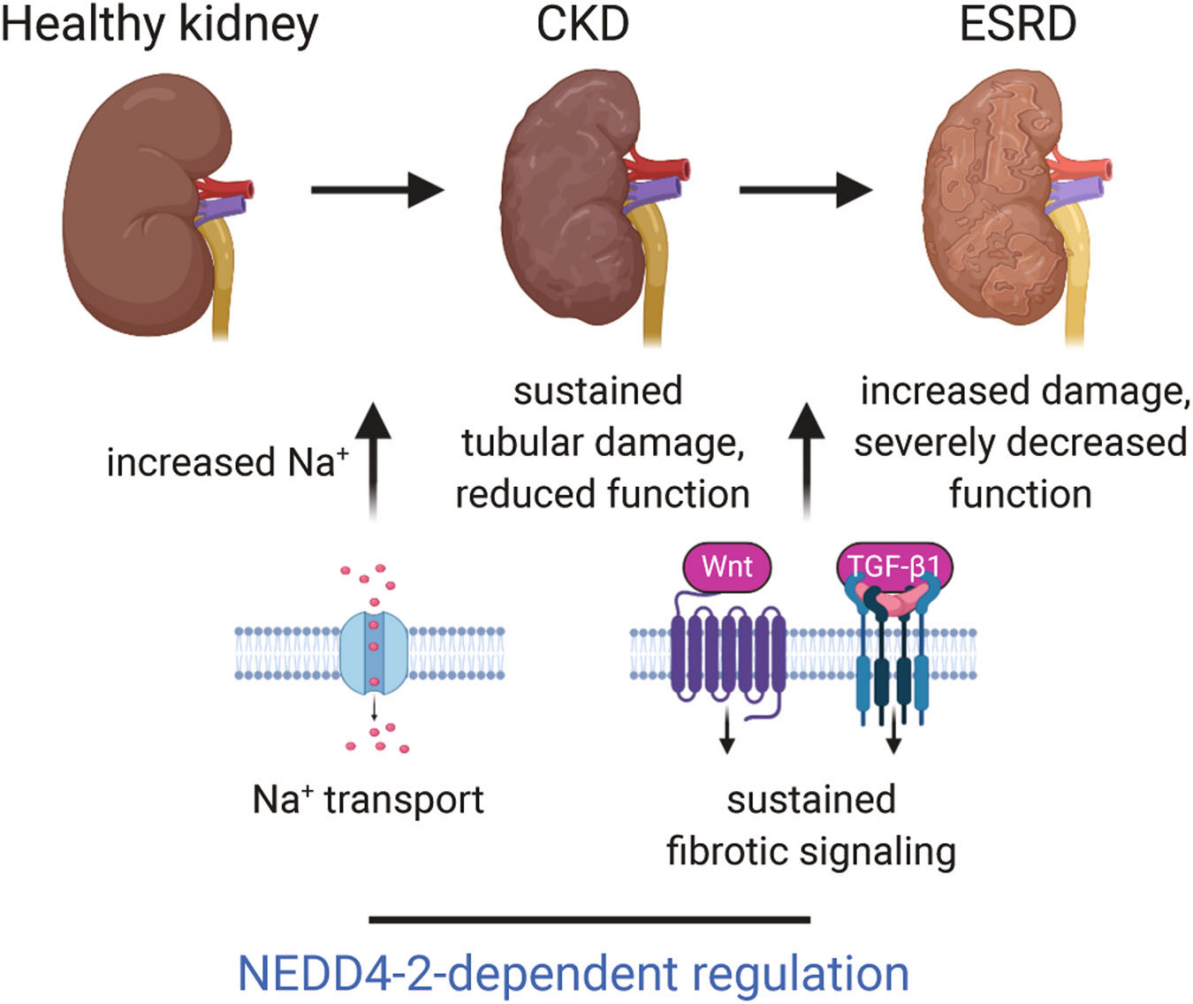
A model of NEDD4-2-dependent control of kidney disease progression. NEDD4-2 regulates several molecular processes that contribute to the progression of kidney disease, including Na^+^ transport and fibrotic signaling pathways. This occurs through ubiquitination of substrates such as ENaC (and other channels and transporters) as well as downstream components of Wnt/β-catenin/TGF-β1 signaling pathways. In the absence of NEDD4-2, increased Na^+^ contributes to sustained tubular damage with a mild reduction in kidney function. Sustained signaling through Wnt and TGF-β1 pathways, particularly as a result of high dietary Na^+^, drives increased kidney damage and severely decreased renal function suggesting progression from CKD to ESRD. Figure created with BioRender.com.

Variants of human *NEDD4L* are associated with renal disease in humans^28^ and loss of *Nedd4-2* in mice results in CKD-like pathology^8^. In this study, we demonstrate that high dietary Na^+^ fed to adult *Nedd4-2*^*Ksp1.3*^ mice drives disease toward ESRD, associated with increased Wnt/β-catenin/TGF-β signaling and resulting in renal damage and fibrosis (Fig. 8). *Nedd4-2* KO in CCD cells confirms the direct involvement of NEDD4-2 in regulating Wnt/β-catenin/TGF-β signaling as well as the role of NEDD4-2 in EMT, potentially contributing to the phenotype in mice.

GFR and other parameters of renal function were significantly altered by high dietary Na^+^ in *Nedd4-2*^*Ksp1.3*^ mice. Together with the weight loss and lethargy of these animals, this is indicative of progression towards ESRD. Increased Na^+^ has been shown to produce a significant rise in BP which can subsequently induce renal damage^29^. However renal damage can be exacerbated by high Na^+^ in the absence of hypertension^30,31^. Given that hypertension is common in several models of *Nedd4-2* deficiency^6^ and *NEDD4L* polymorphisms in humans^6^, it was hypothesized that high dietary Na^+^ may exacerbate kidney damage via increased BP in *Nedd4-2*^*Ksp1.3*^ mice. In this study, although a trend towards increased BP in *Nedd4-2*^*Ksp1.3*^ mice on standard Na^+^ was evident, high Na^+^ diet feeding resulted in decreased BP. This eliminates the possibility that exacerbated kidney disease is caused by Na^+^-induced hypertension, but rather may reflect volume depletion caused by osmotic diuresis. Both polydipsia and polyuria in *Nedd4-2*^*Ksp1.3*^ mice were exacerbated by high Na^+^, reminiscent of nephrogenic diabetes insipidus, often associated with kidney damage and mutations in aquaporin water channels^32–34^. Indeed, *Nedd4-2*^*Ksp1.3*^ kidneys displayed a reduction in the water channels AQP2 and AQP3. Hence, osmotic diuresis and subsequent volume and hemodynamic changes are likely to contribute to kidney damage in *Nedd4-2*^*Ksp1.3*^ mice.

High dietary Na^+^ led to increased areas of vimentin-positive interstitial ECM deposition throughout the renal parenchyma. Vimentin has also been observed in injured tubular cells during tubular degeneration, and during regeneration in acute injury^35,36^; however, was not observed in tubules of *Nedd4-2*^*Ksp1.3*^ kidneys. Interestingly, E-cadherin staining was elevated in *Nedd4-2*^*Ksp1.3*^ kidneys, with a pronounced basolateral and partial apical localization, in contrast to many models of kidney disease where a reduction of E-cadherin is associated with EMT and fibrosis. This is similar to increased E-cadherin in the ligated kidney following ureteric obstruction, particularly in dilated distal tubules surrounded by inflamed interstitium^37^. Hypotonic stress can act as an activator of mechanical stretch-induced E-cadherin in cells^38^, suggesting that increased tubular pressure may contribute to elevated E-cadherin. Furthermore, an increase in a 30 kD E-cadherin fragment was also observed, which may reflect apoptotic cells as caspase-3 cleaves the C terminus to leave a fragment of this size^21^.

Transient activation of Wnt/β-catenin signaling has been associated with kidney injury and recovery, whereas sustained activation of this signaling contributes to renal fibrosis and disease progression, via activation of key fibrosis-related genes^14,15,39^. In the *Nedd4-2*^*Ksp1.3*^ model of kidney disease, mRNA expression of *Wnt* ligands were generally increased, with activation of distinct subsets of ligands sustained by high Na^+^, culminating in β-catenin upregulation. This is similar to the sustained activation of *Wnt* ligands observed in the progression of ischemia-/reperfusion-induced AKI to CKD^14^. In vitro, NEDD4-2 has been shown to regulate Wnt signaling by targeting DVL2 for degradation^23^. Although not observed in CCD cells in vitro, levels of DVL2 were also upregulated in the kidneys of *Nedd4-2*^*Ksp1.3*^ mice, further exaggerated by high Na^+^. Hence, whilst DVL2 may also be regulated by other components of the pathway, our work provides evidence of DVL2 regulation by NEDD4-2 in vivo. Inhibition of WNT signaling by several factors has been shown to protect against kidney fibrosis^39^. The identification of a role for NEDD4-2 in regulating in this pathway in the kidney adds to this understanding, contributing to the identification of potential future therapeutic targets to halt CKD progression.

TGF-β1-induced fibrosis is a key feature of progressive renal disease^40^, and high dietary Na^+^ can increase levels of TGF-β1 in the glomerulus and tubules^12^. NEDD4-2 directly binds to TGF-β activated pSMAD2/3 to ubiquitinate and target these proteins for degradation^24^. Conditional KO of *Nedd4-2* in the lung results in elevated TGF-β signaling via increased pSMAD2/3, which culminates in progressive pulmonary fibrosis^41^. Upregulation of pSMAD2 and pSMAD3 in this study implicates this pathway in kidney disease in *Nedd4-2*^*Ksp1.3*^ mice, particularly after high Na^+^.

In addition to ECM deposition, both TGF-β and Wnt/β-catenin signaling have been associated with EMT in kidney disease. High dietary Na^+^ has also been implicated in EMT in rats^42,43^. However, the contribution of EMT to renal fibrosis has been highly debated^35^, and direct evidence of EMT was not observed in *Nedd4-2*^*Ksp1.3*^ kidneys. Nevertheless, KO of *Nedd4-2* in the CCD cell line resulted in an elongated morphology, increased vimentin and migration rate, and loss of E-cadherin after TGF-β1 stimulation. This implies a partial EMT phenotype after the loss of NEDD4-2 and a sensitivity of these cells to EMT induction by TGF-β1. Importantly, low dietary Na^+^ ameliorated kidney disease in *Nedd4-2*^*Ksp1.3*^ mice, due at least in part to repressed Wnt/β-catenin/TGF-β signaling. Thus, TGF-β and Wnt/β-catenin signaling is important in the Na^+^-induced progression of renal disease, and in parenchymal damage caused by fibrosis, inflammation and tubular cell death. In conclusion, we propose that NEDD4-2 prevents CKD and its progression to ESRD via targeting key proteins involved in Na^+^ homeostasis and fibrotic signaling (Fig. 8). Given the growing association of NEDD4L variants and expression levels with human kidney diseases, this study sheds understanding on how known signaling pathways may be aberrantly regulated in disease and provides a novel potential diagnostic and therapeutic target.

## Materials and methods

### Ethics approval

All animal studies were approved by the institutional ethics and biosafety committees of the University of South Australia and were carried out according to the National Health and Medical Research Council of Australia guidelines.

### Mouse lines and sample collection

Kidney-specific *Nedd4-2*-deficient mice (*Nedd4-2*^*Ksp1.3*^*)* were generated in our laboratory previously^8,44^ and bred at the University of South Australia core animal facility (Adelaide, Australia) under specific pathogen-free conditions. Male mice at 6–8 weeks of age were fed standard sodium chow (0.2% Na^+^), high sodium chow (3.1% Na^+^) or low sodium chow (0.05% Na^+^) (Specialty Feeds, WA, Australia) for 17 days. At the time of collection, after the final metabolic cage, mice were anesthetized, blood collected by cardiac puncture, and organs dissected after cervical dislocation. Capsules were removed from the kidneys and placed into a Histochoice reagent (ProSciTech, Kirwan, QLD, Australia) for histological analysis of paraffin-embedded or frozen samples. For paraffin samples, kidneys were transferred to 70% ethanol and then embedded in paraffin. Kidneys for frozen sectioning were soaked in 30% sucrose overnight before being embedded in OCT (ProSciTech, Kirwan, QLD, Australia). The remaining kidney was snap-frozen in liquid nitrogen for immunoblot or mRNA analysis. Nine mice of each genotype, for each diet condition, were analyzed.

### Histological analysis

Sections (5 μm) were cut using a paraffin microtome, de-paraffinized with xylene, and dehydrated through a graded series of ethanol. Slides were stained with Hematoxylin and Eosin using standard protocols. To evaluate collagen deposition using picrosirius red, slides were stained for 1 h in saturated picric acid with 0.1% Direct Red 80 (Sigma-Aldrich), then washed in 0.01 N hydrochloric acids for 2 min. Digital images were acquired by using a NanoZoomer (Hamamatsu).

### BP measurements

BP was measured by a tail-cuff system (CODA Monitor; Kent Scientific Corporation, Torrington, CT, USA) essentially as described previously^8^. Briefly, at the same time each morning, BP was recorded for 5 acclimation cycles followed by 30–35 measurement cycles. The three highest and lowest readings were removed and an average of the remaining recordings used to calculate the daily BP. An average of 3 days was used to calculate the BP at day 0 before the diet was changed, and an average of BP at post-diet days 13, 14, and 16 used to calculate the final BP. Heating pads were used to keep the mice warm throughout the experiment to ensure sufficient blood flow to the tail.

### Blood and urine analyses

Electrolytes and other parameters of kidney function in blood were carried out by SA Pathology (Adelaide, Australia). Urine osmolality was measured using an Advanced 3320 osmometer (Advanced Instruments) and other parameters using an Advia 2400 chemistry system (Siemens). Serum aldosterone levels were measured using an Aldosterone Elisa Kit (Abcam, ab136933) and analyzed on a Spark 10 M microplate reader (Tecan, Switzerland).

### Metabolic cage studies

Mice were housed in metabolic cages for a 6 h training session, followed by a 24 h session the following day. Mice were provided *ad libitum* access to deionized drinking water and pelleted chow. At the end of the experiment, urine was collected, and blood and tissue samples were taken as described above.

### Generation of *Nedd4-2* KO CCD cell lines

Single guide RNA targeting a region in exon 10 of mouse *Nedd4-2* was cloned into the Px459 vector (Addgene), following the Zhang Genome engineering CRISPR–Cas9 protocol^45^. The following primers were used: F: 5′ CACCGACCGACGCTTCCGCTCTCGG 3′, R: 5′ AAACCCGAGTGCGGAAGCGTCGGTC 3′. The plasmid was transfected using Lipofectamine 3000 reagent (Invitrogen), with an empty Px459 vector serving as a control to generate wild-type clones. After 24 h, media was replaced and supplemented with 2.5 μg/mL puromycin (Sigma-Aldrich) for a further 24 h to select for transfected cells. Surviving cells were passaged at low seeding density and single colonies selected and propagated. Deletion of *Nedd4-2* was confirmed by sequencing of the region in exon 10 and immunoblotting for NEDD4-2 protein. Four clonal cell lines were selected for analysis for both NEDD4-2 KO and wild-type (empty Px459 vector) controls.

### Cell studies

CCD-N21 cells were grown in DMEM/F12 media (Gibco) supplemented with 2% fetal calf serum, 1% ITS, 1 nM 3,3′,5-triiodo-L-thyronin, 10 ng/mL EGF and 50 nM dexamethasone (all from Sigma-Aldrich). Cells were grown at 37 °C in a humidified atmosphere of 5% CO_2_ and passaged twice a week. For experiments in which cells were treated, 1 × 10^5^ cells per well were seeded in 6-well plates for 72 h, or 6 days, before collection and grown in the presence or absence of 2.5 ng/ml recombinant mouse TGF-β1 (R&D Systems, 7666-MB), 10 mM LiCl (Sigma-Aldrich, 746460) or 500 nM LY-364947 (Sigma-Aldrich, L6293). For subcellular fractionation, cells were lysed in ice-cold cytoplasmic extraction buffer (10 mM HEPES, 1.5 mM MgCl_2_, 10 mM KCl, 0.5 mM DTT, 0.05% NP40, and HALT protease and phosphatase inhibitor cocktail [Thermo Fisher Scientific] pH 7.9) for 10 min on ice, centrifuged at 3000 rpm for 10 min and supernatant reserved. The nuclear protein-containing pellet was then resuspended in ice-cold nuclear extraction buffer (5 mM HEPES, 1.5 mM MgCl_2_, 300 mM NaCl, 0.2 mM EDTA, 0.5 mM DTT, and 26% glycerol, pH 7.9), sonicated, and left on ice for 30 min before centrifuging at 16,000 rpm for 20 min with supernatant reserved. For wound closure (scratch) assay, cells were grown to full confluency before a vertical scratch in the cell layer was performed using a 20 μl pipette tip. Cells were washed with fresh media added and images were taken at 0, 8, and 24 h post scratch. The average distance migrated from each leading edge was measured for each time point.

### Immunoblotting

For mouse samples, half of each kidney was lysed in ice-cold extraction buffer at pH 7.5 (50 mM Tris-HCl pH7.5, 1 mM EDTA, 1 mM EGTA, 0.27 M sucrose, 0.1% β-mercaptoethanol and HALT protease and phosphatase inhibitor cocktail [Thermo Fisher Scientific]). Tissue was homogenized, frozen in liquid nitrogen, immediately thawed, and incubated at 4 °C on a nutator for 30 min and centrifuged at 13,000 rpm for 5 min. For cell studies, cells were lysed in Verhagen lysis buffer (150 mM NaCl, 20 mM Tris-HCl pH 7.5, 2 mM EDTA, 10% glycerol, and 1% Triton-X) and lysed as above. For pSMAD2/3 immunoblots, cells were lysed in sodium dodecyl sulfate (SDS) lysis buffer (150 mM NaCl, 10 mM Tris-HCl pH 8.0, 2% SDS), boiled for 5 min then centrifuged at 13,000 rpm for 5 min. Supernatant protein (25 μg) was combined with protein load buffer (100 mM Tris-HCl pH 6.8, 200 mM DTT, 4% SDS, 0.2% bromophenol blue, 20% glycerol), heated at 37 °C for 30 min, loaded onto 4–20% precast sodium dodecyl sulfate-polyacrylamide gel electrophoresis gels (Bio-Rad) and transferred to PVDF membrane using the Trans-blot Turbo instrument (Bio-Rad). Membranes were blocked with 5% skim milk in TBS-T (Tris-buffered saline, 0.05% Tween 20) and primary antibodies added; anti-AQP2 (Abcam, ab65837, 1:1000), anti-AQP3 (Abcam, ab125219, 1:1000), anti-vimentin (Abcam, #ab92547, 1:1000), anti-αSMA (Abcam, ab7817, 1:2000), anti-E-cadherin (Cell Signaling Technology, #24E10, 1:1000), anti-GAPDH (Cell Signaling Technology, 14C10, 1:10000), anti-β-catenin (Cell Signaling Technology, 6B3, 1:1000), anti-pSMAD2 (Thermo Fisher, 44244 G, 1:1000), anti-pSMAD3 (Abcam, ab52903, 1:1000), anti-SMAD2/3 (Cell Signaling Technology, 8685P, 1:1000), anti-TGF-βR1 (Invitrogen, PA5-32631, 1:1000), anti-DVL2 (Cell Signaling Technology, 3216, 1:1000), anti-NEDD4-2 (in house^46^, 1:1000), anti-α-Tubulin (Abcam, ab4074, 1:1000), anti-H3 (Abcam, ab176842, 1:1000), anti-β-actin (Sigma, AM4302, 1:2000), anti-N-cadherin (Cell Signaling Technology, 14215, 1:1000). Horseradish peroxidase secondary antibodies (Millipore) was added at 1:2000 and developed with ECL Prime (GE Healthcare) or West Femto (Thermo Scientific). GAPDH was developed using Cy5 secondary antibody (GE Healthcare). Images were acquired on a ChemiDoc Touch Imager (BioRad). Quantitation was conducted using Image Lab Software (BioRad), with each band normalized to GAPDH or β-actin.

### Immunostaining

Paraffin sections (5 μm) were deparaffinized and hydrated in a graded ethanol series. Heat-mediated antigen retrieval was carried out by boiling for 10 min in 10 mM citric acid solution (pH 6). Tissue sections were blocked with 10% goat serum. Primary antibodies used were as above (all at 1:200): AQP2, AQP3, Vimentin, and E-cadherin. Additional antibodies were β-catenin (BD Transduction Labs, 610154); rhodamine-labeled DBA (Vector Laboratories, RL-1032); FITC-conjugated mouse anti-αSMA (Sigma-Aldrich, F3777). For immunostaining of cells, cells were grown on coverslips and fixed for 15 min in 4% PFA, then blocked for 1 h in 1% BSA with 0.5% Triton-X. Sections were incubated with primary antibody (as above) and Alexa Fluoro 594 Phalloidin (Thermo Fisher, A12381) overnight at 4 °C and with secondary for 1 h at room temperature.

Sections were then incubated with the corresponding fluorescently tagged secondary antibody (AlexaFluor-488 or AlexaFluor-568, Thermo Fisher Scientific), counterstained with Hoechst 33342 (Thermo Fisher) and mounted in Prolong Gold Antifade reagent (Invitrogen). Stained samples were imaged using an LSM 800 confocal microscope using Zen 2011 (Black Edition) version 8.1.5.484 (Carl Zeiss Microscopy, Jena, Germany). Image analysis was conducted using Adobe image suite software.

### Quantitative real-time PCR

Total RNA was isolated from half of each kidney using TRIzol Reagent (Life Technologies) and RNA was reverse-transcribed with a High Capacity cDNA reverse transcription kit (Applied Biosciences). qRT-PCR was performed and analyzed as described^47^, where all data are normalized to *TBP* (*TATA box binding protein*) levels. Primer sequences are *Collagen-1* (*Col1a1*) F: CGGAGAAGAAGGAAAACGAGGAG, R: CACCATCAGCACCAGGGAAAC. *Vimentin*, F: CGGCTGCGAGAGAAATTGC, R: CCACTTTCCGTTCAAGGTCAAG. *Kidney injury molecule 1* (*Kim-1*), F: TGGTTGCCTTCCGTGTCTCT, R: TCAGCTCGGGAATGCACAA, *Wnt1*, F: TCAGAACCGCAGCACAGAAC, R: TTCACGATGCCCCACCATC. *Wnt3*, F: GGGGCGTATTCAAGTAGCTG, R: GTAGGGACCTCCCATTGGAT. *Wnt4*, F: CGAGCAATTGGCTGTACCTGG, R: CAGGCCTTTGAGTTTCTCGC. *Fibronectin*, F: GAAGACAGATGAGCTTCCCCA, R: GGTTGGTGATGAAGGGGGTC. *Serpine*, F: ACAACCCGACAGAGACAATCC, R: TCGTCCCAAATGAAGGCGTC. *Twist*, F: GAGGTCTTGCCAATCAGCCA, R: CCAGTTTGATCCCAGCGTTT. *cMyc*, F: TGTTCTCTGCCTCTGCCCG, R: GCATCGTCGTGGCTGTCTG. *Cyclin D1*, F: CCTCTCCTGCTACCGCACAA, R: TTGTTTAGCCAGAGGCCGGT. *TGF-β1*, F: GATACGCCTGAGTGGCTGTC, R: AAGCCCTGTATTCCGTCTCC. *TATA-box binding protein* (*TBP*), F: CAAACCCAGAATTGTTCTCCTT, R: ATGTGGTCTTCCTGAATCCCT.

### Statistical analysis of data

Statistical analysis was performed using GraphPad Prism software (v6.0). A Mann–Whitney test for non-parametric data was used to assess changes in blood/urine parameters in Tables 1 and 2, and one-way ANOVA used to assess repeated measurements for BP and weight changes. The remaining data were analyzed using an unpaired two-tailed Student’s *t* test. A *P* value of ≤0.05 was considered significant. All values are presented as mean ± SEM (or ±SD for immunoblot quantitation), as indicated in the figure legends.

## Supplementary information

Supplementary Information

## Data Availability

The datasets used and/or analyzed during the current study are available from the corresponding author on reasonable request.
