# Supplementary material for: The ubiquitin ligase NEDD4-2/NEDD4L regulates both sodium homeostasis and fibrotic signaling to prevent end-stage renal disease
Source: Cell Death Dis. 2021 Apr 14;12(4):398. doi: 10.1038/s41419-021-03688-7 (PMC8046789; doi:10.1038/s41419-021-03688-7)
Supplement: Supplementary file 1 — Supplementary Information [file 41419_2021_3688_MOESM1_ESM.pdf]

Supplementary Information

This file contains the following Supplementary Information:

Supplementary Fig. 1. Weight change over experimental diet duration
Supplementary Fig. 2. Kidney injury in *Nedd4-2*$^{Ksp1.3}$ mice
Supplementary Fig. 3. Signaling pathways in *Nedd4-2*$^{Ksp1.3}$ mice
Supplementary Fig. 4. Generation of NEDD4-2 KO cells

Supplementary Figure 1

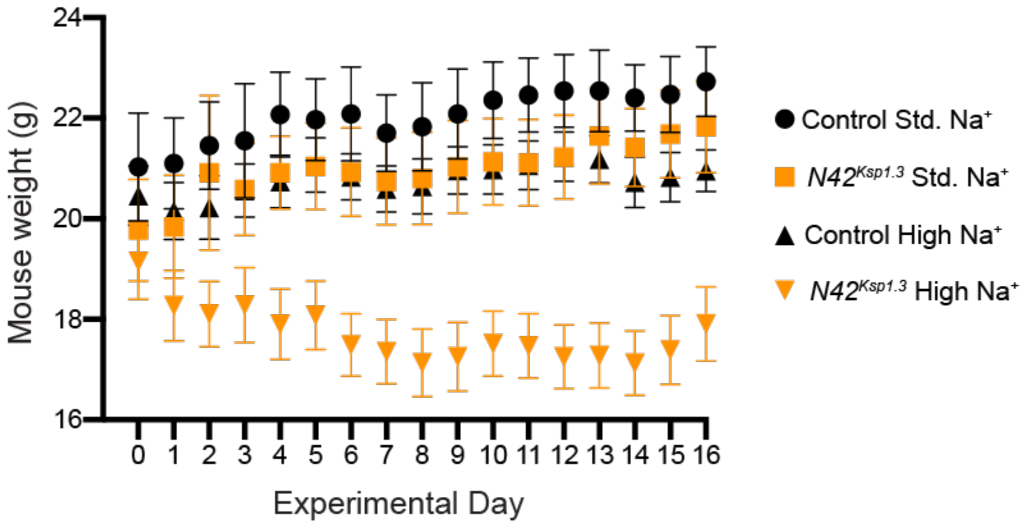

Supplementary Fig. 1. Weight change over experimental diet duration

Daily mouse weight on standard (Std.) or high Na^+ diet, n=7-11. Data presented as means ± SEM.

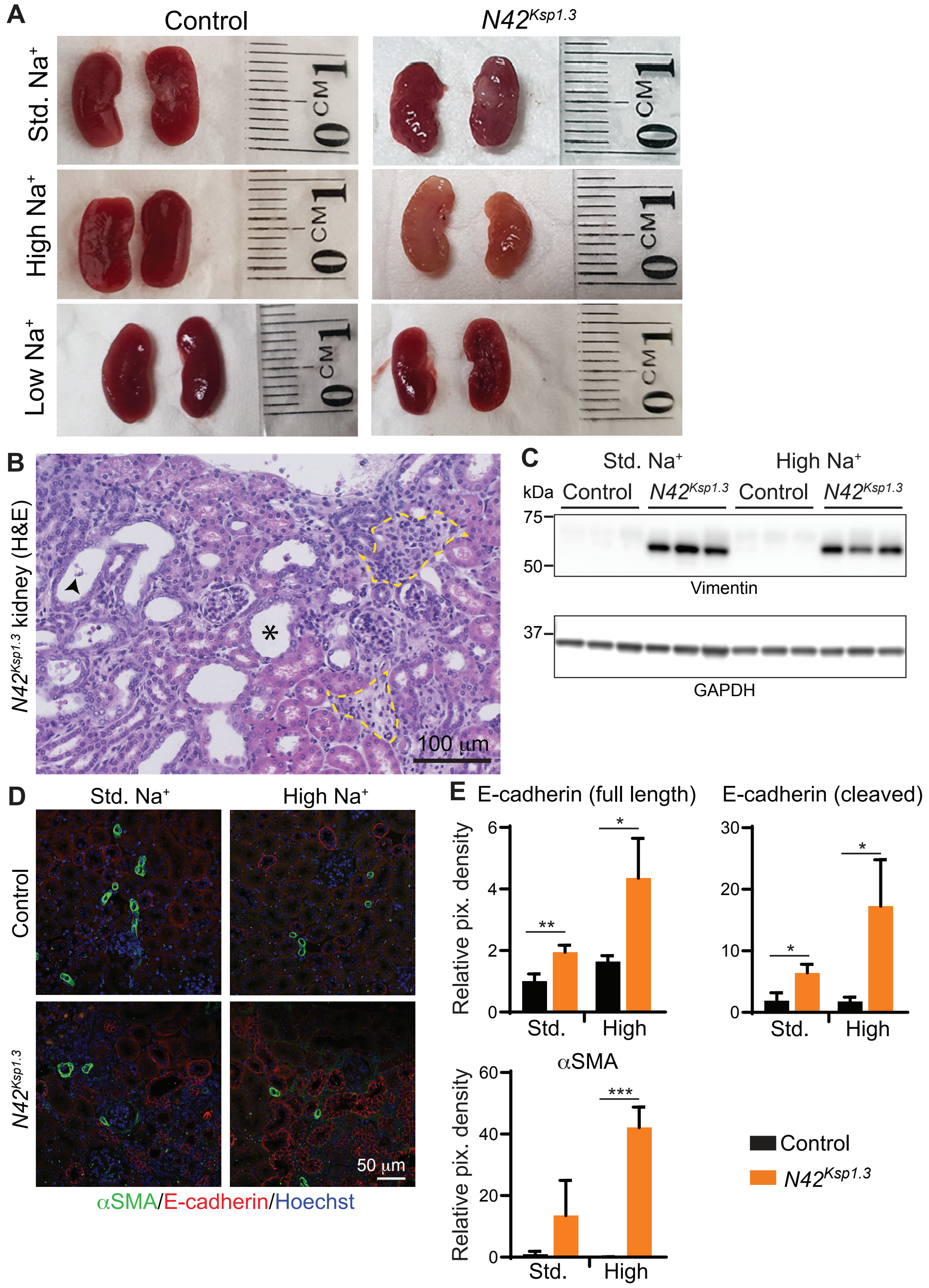

Supplementary Fig. 2. Kidney injury in *Nedd4-2*$^{Ksp1.3}$ mice

A Gross morphology of kidneys from mice fed standard (Std.), high or low Na^+ diets. **B** A representative example of Heamatoxylin and Eosin staining of *Nedd4-2*$^{Ksp1.3}$ kidney after high Na^+ diet. As examples, * indicates dilated tubule, dotted line shows areas of mesenchymal infiltration and arrowhead points to cell debris. Scale bar, 100 µm. **C** Immunoblot analysis of Vimentin with GAPDH as loading control. **D** Lower magnification images from Fig. 3E. **E** Quantitation of immunoblots from Fig. 3F normalized to GAPDH loading control, n=3. Data presented as means ± SD with significance determined using unpaired 2-tailed Student's t-test. *$P < 0.05$, **$P < 0.01$, ***$P < 0.005$.

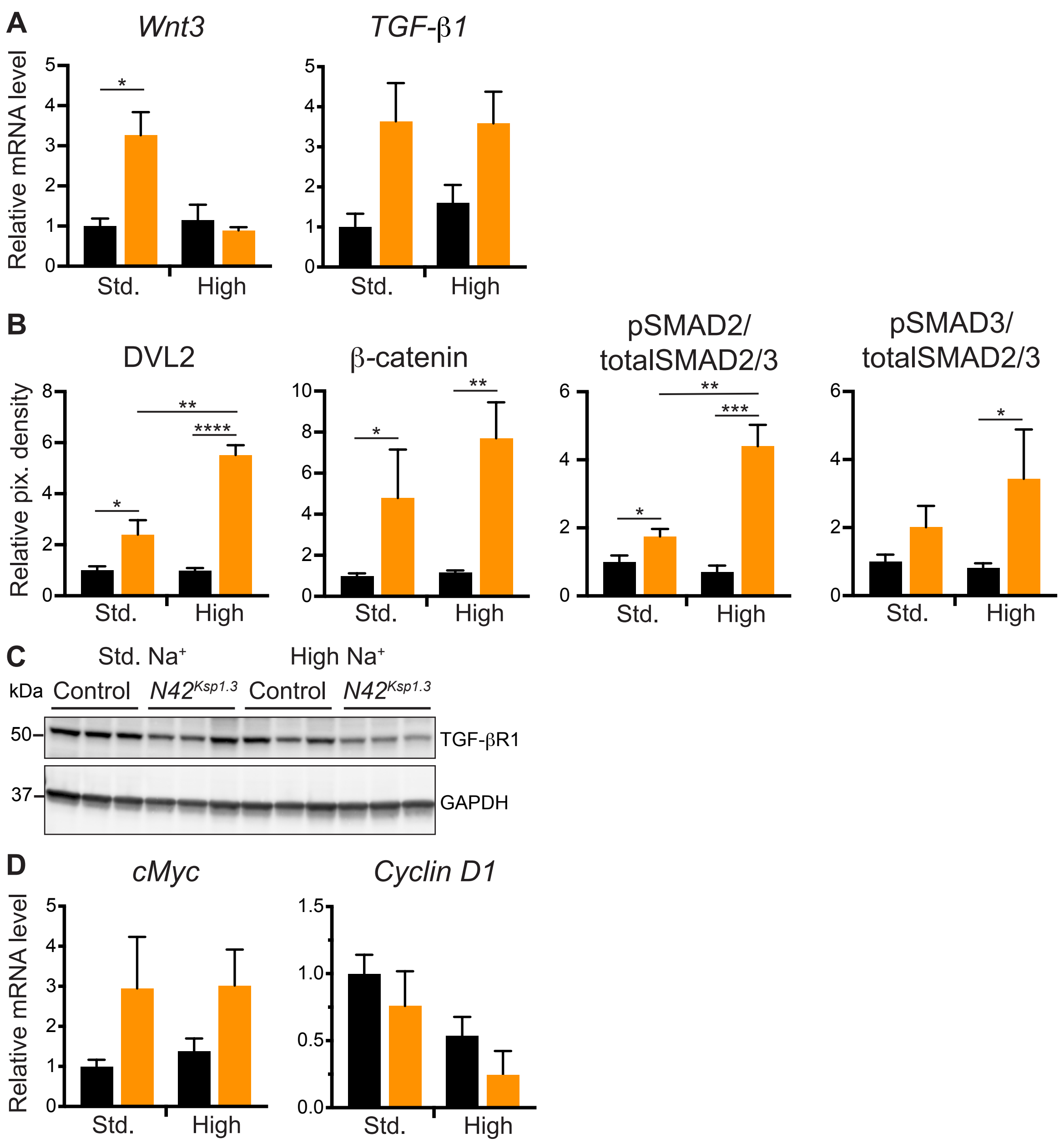

Supplementary Fig. 3. Signaling pathways in *Nedd4-2$^{Ksp1.3}$* mice

A qRT-PCR analysis of *Wnt3* and *TGF-β1* relative to TBP. **B** Quantitation of immunoblots from Fig. 4C normalized to GAPDH loading control, n=3. **C** Immunoblot analysis of TGF-βR1 with GAPDH as loading control. **D** qRT-PCR analysis of *cMyc* and *Cyclin D1* relative to TBP. Data presented as means ± SEM (**A**, **D**) or means ± SD (**B**) with significance determined using unpaired 2-tailed Student's t-test, n = 3-4. **P* < 0.05, ***P* < 0.01, ****P* <0.005, *****P* < 0.001.

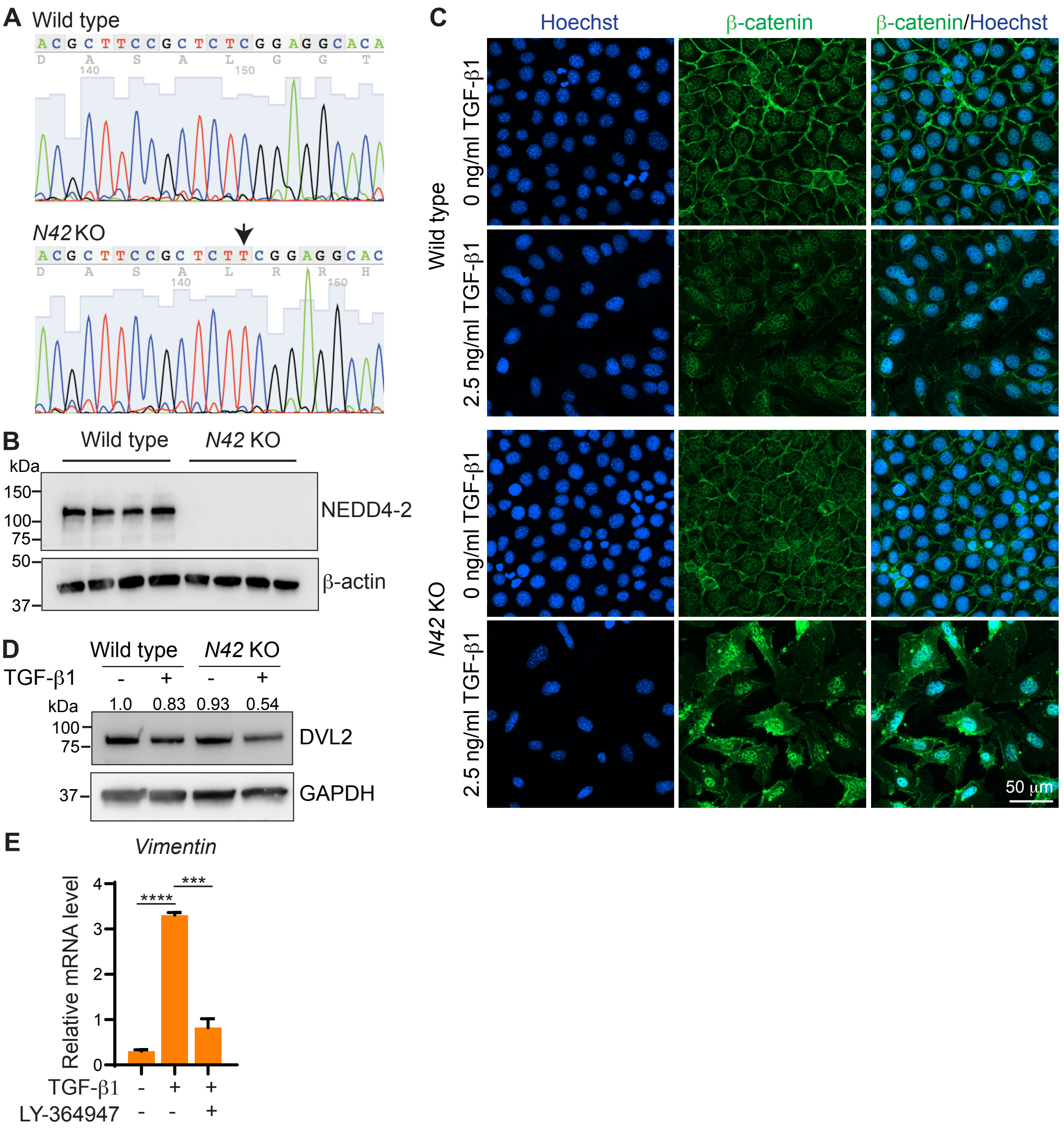

Supplementary Fig. 4. Generation of NEDD4-2 KO cells

A An example of sequencing of a *Nedd4-2* (*N42*) KO clone, showing insertion of a single amino acid. **B** Immunoblot of 4 independent wild type and *Nedd4-2* KO clonal cell lines with β-actin as loading control. **C** Lower magnification and single color panels together with merged images for Fig. 5E of β-catenin and Hoechst staining of 72 h TGF-β1 treated CCD cells. **C** Immunoblot analysis of DVL2 with GAPDH as loading control. Numbers indicate quantitation of DVL2 bands relative to untreated wild type lane, normalized to GAPDH. **E** qRT-PCR analysis of *Vimentin* relative to *TBP* after treatment with TGF-β1 and LY-363947 inhibitor. Data presented as means ± SEM with significance determined using unpaired 2-tailed Student's t-test, n = 4. ***$P < 0.005$, ****$P < 0.001$.
